# Association between blood lead levels and stroke: Evidence from a US National Health Survey

**DOI:** 10.1097/MD.0000000000046815

**Published:** 2026-01-23

**Authors:** Jian Li, Qiujing Li, Lichun Zhou

**Affiliations:** a Department of Neurology, Beijing Chao-Yang Hospital, Capital Medical University, Beijing, China; b Department of Public Laboratory, The Third People’s Hospital of Kunming City/Infectious Disease Clinical Medical Center of Yunnan Province, Kunming, Yunnan, China.

**Keywords:** blood lead concentration (BLC), NHANES (National Health and Nutrition Examination Survey), stroke

## Abstract

Stroke is acknowledged as a principal factor contributing to the overall burden of disability. Nevertheless, there has been little research into the relationship between blood lead concentration (BLC) and stroke. The objective of this study was to assess the potential association between BLC and stroke within the United States. The data from the National Health and Nutrition Examination Survey for the period 2017 to 2018 were collected and subjected to analysis. Stroke cases were identified through the utilization of a question designed to ascertain whether the subject had been informed by a medical practitioner that they had experienced a stroke. A multivariate logistic regression model was employed to calculate the odds ratio (OR) and assess the potential deleterious effects of BLC. The study cohort comprised 5013 individuals with a mean age of 48.47 ± 0.58 years, 48.02% male and 51.98% female. Individuals with elevated BLC levels were more likely to be male (58.17% vs 31.37%, *P* < .0001), aged over 65 (34.16% vs 4.72%, *P* < .0001), non-Hispanic White (62.93% vs 59.43%, *P* < .0001), physically inactive (48.09% vs 44.36%, *P* = .0175), have a lower income-to-poverty ratio (23.21% vs 21.82% for poverty-income ratio < 1.3, *P* = .001), and possess a higher body mass index (BMI). After controlling for potential confounding variables, a significant positive association between BLC and stroke was observed (OR: 4.44, 95% confidence interval [CI]: 1.89–10.4, *P* = .002). The findings suggest that individuals under the age of 65 years (OR: 3.12, 95% CI: 1.51–6.46, *P* = .005), and females (OR: 2.89, 95% CI: 1.53–5.45, *P* = .003) were at particularly elevated risk. The elevated risk of stroke observed in individuals with elevated BLC levels was significant across all ethnic groups, with non-Hispanic Blacks showing the strongest association (OR: 5.39, 95% CI: 1.75–16.57, *P* = .006). The results of this study indicate a positive correlation between BLC and stroke. Furthermore, individuals under the age of 65, females, non-Hispanic Whites, and those with a BMI exceeding 25 kg/m^2^ were found to be significantly associated with an increased risk of stroke.

## 1. Introduction

Globally, approximately 13.7 million individuals are impacted by stroke each year,^[1]^ constituting a predominant cause of disability. It is estimated that one in 4 adults will encounter a stroke at some juncture during their lifetime. It is the second leading cause of mortality, accounting for 5.5 million deaths annually.^[2]^ Ischemic stroke represents the most prevailing type of stroke, exerting a significant influence on the incidence of permanent disability and a notable deterioration in quality of life. This, in turn, gives rise to a considerable burden of disease. In conclusion, the effects are detrimental both to nations and individuals. The occurrence of stroke is regarded as being associated with several risk factors, including smoking, alcohol consumption, hypertension, diabetes, dyslipidemia, and others.^[3]^ It is of the utmost significance to identify the risk factors related to stroke.

Lead is a non-essential metal that can be toxic to human tissues even at low concentrations and is classified as the second most harmful substance by the Toxic Substances and Disease Registry.^[4]^ Despite endeavors to reduce lead exposure, the body burden of lead increases with age. The primary sources of lead contamination encompass paint, water, food, dust, soil, cookware, and leaded gasoline. The majority of cases of lead poisoning can be ascribed to oral ingestion and subsequent gastrointestinal absorption.^[5]^ A considerable portion of ingested lead accumulates in specific target tissues, such as blood, soft tissues, and bones, where it has a long half-life.^[6]^ Chronic exposure to lead has been demonstrated to adversely affect numerous organ systems, including the central nervous system, kidneys, cardiovascular system, reproductive system, and blood system. Additionally, there is evidence suggesting that chronic lead exposure may result in cognitive decline.^[7]^

The pathophysiological mechanisms linking lead exposure to stroke risk involve several interconnected pathways. Lead is known to induce oxidative stress by generating reactive oxygen species, which damage vascular endothelial cells and promote atherosclerosis. Lead exposure also activates inflammatory processes, increasing pro-inflammatory cytokines that contribute to vascular dysfunction. Moreover, lead has been associated with hypertension through its effects on renal function, the renin-angiotensin system, and vascular smooth muscle reactivity. Lead can also alter hemodynamic properties by affecting platelet function and increasing blood viscosity, which may contribute to thrombus formation. These mechanisms collectively increase the risk of both ischemic and hemorrhagic stroke. Despite this biological plausibility, epidemiological evidence directly linking blood lead levels to stroke occurrence remains limited, highlighting the need for further investigation.

Data from the National Health and Nutrition Examination Survey (NHANES) imply that even low levels of lead exposure can have substantial health implications, particularly for vulnerable populations, such as the elderly.^[8]^ Previous studies have mainly focused on the relationship between blood lead levels and diseases like hypertension, macular degeneration, and atherosclerosis, with the majority of the study populations consisting of women, children, patients with chronic obstructive pulmonary disease, and diabetic patients.^[9]^

This study aims to explore the correlation between blood lead levels and the incidence of stroke, providing feasible evidence to support stroke prevention and highlighting the significance of monitoring and reducing lead exposure in the population. Through meticulous analysis of the association between blood lead levels and stroke incidence, this research will offer a scientific basis for formulating effective public health policies to address the burden of stroke related to lead exposure.

## 2. Methods

### 2.1. Study population

The data for the present study were obtained from the NHANES, which was conducted during the 2017 to 2018 period. The NHANES is a nationally representative cross-sectional study designed to examine demographic, socioeconomic, health nutritional information.^[10]^ Our research is based on data from the NHANES and examines the relationship between blood lead levels and stroke among all surveyed populations from 2017 to 2018. Understanding the mechanisms linking blood lead concentration (BLC) to the occurrence of stroke is indispensable for developing effective public health strategies to reduce lead exposure and its related health risks.

### 2.2. Inclusion and exclusion criteria

The inclusion criteria for this study were: participants aged 18 years and older; participants with available BLC measurements; and participants who completed the questionnaire regarding stroke history. The exclusion criteria were: individuals younger than 18 years; participants with missing data on self-reported stroke status; participants lacking BLC measurements; and participants with incomplete data on weight or essential covariates that would affect our analysis.

The cross-sectional nature of NHANES means that participants were not followed over time; rather, data was collected during a single examination period in 2017 to 2018. The NHANES program employs a complex, multistage probability sampling design to select participants representative of the civilian, non-institutionalized US population. The sample size determination for NHANES is conducted by the National Center for Health Statistics to ensure adequate statistical power for detecting meaningful associations while maintaining representativeness of the US population.

**Figure 1.**
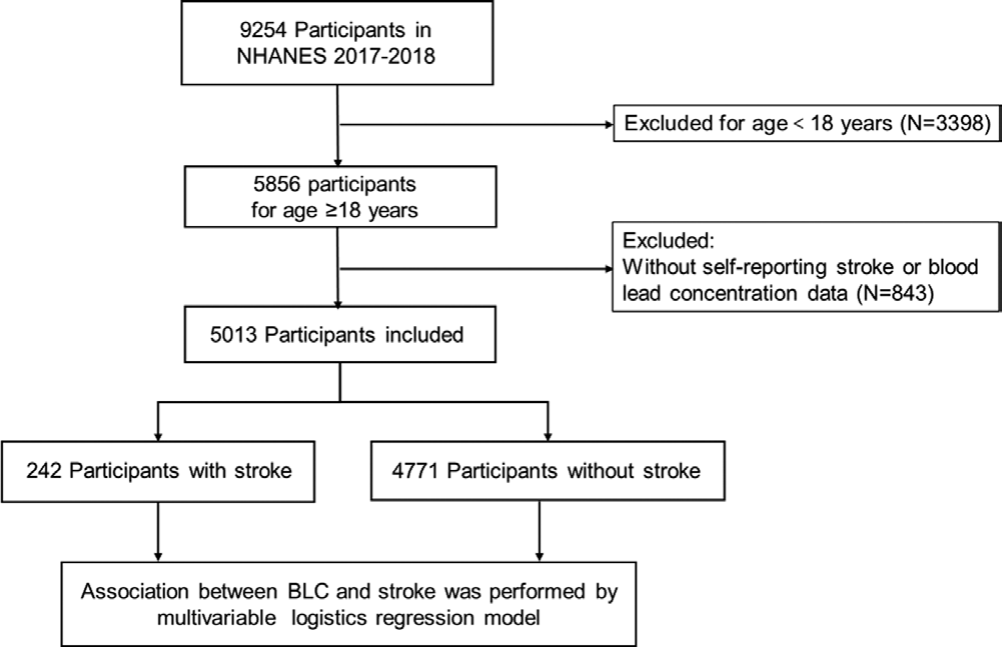
A flowchart showing the selection of study participants. BLC = blood lead concentration, NHANES = National Health and Nutrition Examination Survey.

Individuals below the age of 18 years and those without self-reported data on stroke or lead were excluded. Furthermore, participants with incomplete data regarding weight or covariates were excluded. Consequently, 5013 participants were included in the final analysis (Fig. 1). Written informed consent was obtained from all study participants, and the study protocols were approved by the Research Ethics Review Board of the National Centre for Health Statistics. However, informed consent was not required for secondary analysis of publicly available data. This report was also produced following the Strengthening the Reporting of Observational Studies in Epidemiology guidelines.^[11]^

### 2.3. Primary exposure

The lead concentration of the whole blood was chosen as the exposure biomarker and was measured using inductively coupled plasma dynamic reaction cell mass spectrometry (ICP-DRC-MS) after a simple dilution sample preparation step.^[12]^ Detailed information on laboratory quality assurance and monitoring is available on the NHANES website. BLC were categorized into evenly distributed tertiles (≤0.02 µmol/L, 0.03–0.05 µmol/L, ≥0.06 µmol/L).

### 2.4. Outcome

The study outcome of interest, the occurrence of stroke, was determined with the survey question “Has a doctor or other health professional ever told you that you had a stroke” from the “medical conditions” section of the questionnaire. In addition, 5013 participants who were older than 18 years answered the question. Notably, 242 individuals answered “Yes” while 4771 participants answered “No.”

### 2.5. Covariates

Covariates were determined based on existing knowledge and literature. Age, gender, and race/ethnicity were the first factors to be considered.

In this study, age was classified into 2 categories: ≤65 and >65 years. Race/ethnicity was classified as Mexican American, other Hispanic, non-Hispanic White (whites without Hispanic ancestry), non-Hispanic Black (black without Hispanic ancestry), and other races (non-Hispanic Asian, Native Alaskans, American Indians, Native Hawaiians or other Pacific Islanders, and multiracial people). According to the Supplemental Nutrition Assistance Program recommended method, we divided the population into 3 groups according to the poverty income ratio (PIR): 0 to 1.3, 1.3 to 3.5, and ≥3.5, which represent 3 different family economic levels, and the higher the PIR, the better the economic condition.

By interviewing the participants, the educational level was also determined, including more than high school, high school, and less than high school. Smoking was classified according to the serum cotinine levels into low (<0.015 ng/mL), moderate (0.015–3 ng/mL), and high levels (>3 ng/mL). Alcohol consumption was classified as never, moderate (1 drink/day for women or 1 to 2 drinks/day for men), or heavy (≥2 drinks/day for women or ≥3 drinks/day for men) based on criteria of the National Institute on Alcohol Abuse and Alcoholism.

Additionally, based on Physical Activity Guidelines recommendation of ≥75 min/week of vigorous or ≥150 min/week of moderate physical activity, participants were classified into 3 groups, namely active (≥the level of recommended activity), less active (<the level of recommended activity), and inactive (no physical activity). Depending on body mass index (BMI), people were classified into underweight (<20.0 kg/m^2^), normal weight (20–25.0 kg/m^2^), overweight (25.0–30.0 kg/m^2^), or obese (≥30.0 kg/m^2^).

### 2.6. Statistical analysis

We analyzed both continuous and categorical variables in relation to BLC and stroke outcomes. For demographic characteristics, we examined age, gender, race/ethnicity, education level, and PIR. Health-related variables included BMI, physical activity level, smoking status (using serum cotinine as a biomarker), alcohol consumption, and history of diabetes. We also analyzed biochemical parameters including total cholesterol, triglycerides, and heavy metals (chromium, mercury, selenium, manganese, and lead).

All analyses incorporated the complex survey design of NHANES, including stratification, clustering, and sampling weights, to ensure nationally representative estimates. For the primary analysis examining the association between BLC and stroke, we constructed 3 logistic regression models with progressive adjustment for potential confounders: Model 1 (unadjusted), Model 2 (adjusted for demographic factors), and Model 3 (fully adjusted for all covariates). We also conducted stratified analyses to examine potential effect modification by age, gender, race/ethnicity, and BMI categories.

Continuous variables were described as weighted mean ± standard error and compared using weighted linear regression. Categorical variables were expressed as weighted percentages (95% confidence interval [CI]) and compared using the χ^2^ test. Multivariable logistic regression models were constructed to assess the association between BLC and stroke. Most or all of these confounders were accounted for in the final model, including age group, gender, race/ethnicity, the PIR, level of education, serum cotinine levels, daily alcohol consumption, history of diabetes, physical activity status, BMI groups, diabetes, triglycerides, total cholesterol, and other heavy metals.

The statistical analyses were conducted using STATA 17.0 (StataCorp, College Station) and EmpowerStats (version 2.0). Two-sided *P* < .05 was considered statistically significant.

## 3. Results

### 3.1. Overall characteristics of the participants

A total of 5013 participants with BLC ranging from 0 to 2.05 µmol/L. The study population with an average age of 48.47 years old, males account for 48.02%. Among all participants, the prevalence of stroke was 3.28%, and it increased with the higher BLC tertiles. The clinical characteristics of the participants by BLC tertiles are shown in Table 1, from which we can find statistically significant differences in age, gender, race, BMI, physical activity level, PIR, education, smoking, alcohol use, history of stroke, total cholesterol, triglyceride, chromium, hydrargyrum, selenium, and plumbum (all *P* < .05).

**Table 1:** Characteristics of participants (n = 5013) stratified by blood lead concentration tertiles (μmol/L) in NHANES 2017–2018.

Variable	Total	Tertile 1 (≤0.02)	Tertile 2 (0.03–0.05)	Tertile 3 (≥0.06)	*P*-value
Age, yr (mean ± SE)	48.47 ± 0.58	37.09 ± 0.46	47.92 ± 0.85	57.20 ± 0.64	<.001
Age group, %					
≤65 yr	79.47	95.28	81.43	65.84	<.001
>65 yr	20.53	4.72	18.57	34.16	
Gender, %					
Male	48.02	31.37	49.30	58.17	<.001
Female	51.98	68.63	50.70	41.83	
Race/ethnicity, %					
Hispanic	15.97	22.27	15.37	12.27	<.001
Non-Hispanic White	62.64	59.43	64.21	62.93	
Non-Hispanic Black	10.97	11.39	10.50	11.27	
Other race	10.42	6.91	9.92	13.52	
Socioeconomic factors					
Education, %					
More than high school	61.41	65.17	64.73	54.59	<.001
High school or equivalent	27.21	26.07	25.63	29.99	
Less than high school	11.27	8.76	9.52	15.25	
Poverty-income ratio, %					
<1.3	21.08	21.82	18.95	23.21	.001
1.3–3.5	28.69	30.22	28.44	27.91	
>3.5	39.60	37.60	42.74	37.10	
Health behaviors & status					
BMI, kg/m^2^ (mean ± SE)	29.83 ± 0.32	30.46 ± 0.46	30.14 ± 0.39	28.98 ± 0.38	.004
BMI group, %					
<20	4.61	4.71	4.69	4.45	<.001
20–25	20.76	21.67	18.52	22.91	
25–30	31.16	23.79	31.40	36.08	
>30	42.43	49.11	44.62	34.96	
Physical activity level, %					
Inactive	47.38	44.36	48.51	48.09	.018
Less active	8.54	9.36	8.85	7.58	
Active	43.79	46.23	42.42	43.78	
Smoking (serum cotinine), %					
Low (<0.015 ng/mL)	37.81	42.70	39.08	32.76	<.001
Moderate (0.015–3 ng/mL)	36.77	41.71	34.60	36.01	
High (>3 ng/mL)	24.28	13.90	25.42	30.19	
Medical history					
History of diabetes, %					
No	85.95	87.13	84.86	86.46	.148
Yes	14.05	12.87	15.14	13.54	
History of stroke, %					
No	96.72	98.74	97.34	94.52	<.001
Yes	3.28	1.26	2.66	5.48	
Laboratory parameters					
Total cholesterol, mmol/L	4.90 ± 0.05	4.69 ± 0.06	4.87 ± 0.05	5.08 ± 0.06	<.001
Triglycerides, mg/dL	1.63 ± 0.04	1.55 ± 0.06	1.63 ± 0.06	1.68 ± 0.05	.018
Blood lead, μmol/L	0.05 ± 0.00	0.02 ± 0.00	0.04 ± 0.00	0.10 ± 0.00	<.001

Values are weighted mean ± standard error (SE) or weighted percentage. *P* values are for differences across tertiles of blood lead concentration.

BMI = body mass index, NHANES = National Health and Nutrition Examination Survey.

The mean BLC for all participants was 0.05 µmol/L, with the values for the different tertiles as follows: tertile 1: ≤0.02; tertile 2: 0.03 to 0.05; and tertile 3: ≥0.06. Compared to those with the lowest tertile of BLC, individuals with the highest tertile of BLC were more likely to be male (58.17% vs 31.37%, *P* < .0001), older (mean age 57.20 vs 37.09 years, *P* < .0001), non-Hispanic White (62.93% vs 59.43%, *P* < .0001), physically inactive (48.09% vs 44.36%, *P* = .0175), had lower PIR (23.21% vs 21.82% for PIR < 1.3, *P* = .001), and a higher BMI. The level of education also affected BLC, as those with more education have significantly lower BLC than those with less education (54.59% vs 65.17% for more than high school, *P* < .0001). In addition, subjects with increased BLC had higher total cholesterol (5.08 vs 4.69 mmol/L, *P* < .0001) and triglyceride levels (1.68 vs 1.55 mg/dL, *P* = .0182).

### 3.2. Associations between BLC and stroke

The multivariate logistic regression results are shown in Table 2. In the initial risk analyses, we found significant associations between BLC and the prevalence of stroke. As shown in Table 2, after full adjustment for covariates, compared with individuals with BLC in the lowest tertile (0–0.02 µmol/L), the odds ratio (OR) for risk of stroke was 3.12 (95% CI: 1.51–6.48, *P* = .005) for individuals with concentrations of BLC in the highest tertile (≥0.06 µmol/L) in the fully adjusted model.

**Table 2:** Associations between blood lead concentration and stroke (n = 5013), NHANES 2017–2018.

	Stroke	Model 1	Model 2	Model 3
	(Yes, n = 242)	OR (95% CI), *P*	OR (95% CI), *P*	OR (95% CI), *P*
BLC	242	5.03 (1.17–21.66), .033	4.16 (1.69–10.24), .004	4.44 (1.89–10.4), .002
Tertiles of BLC, nmol/L		Reference	Reference	Reference
T1	17	2.15 (1.1–4.19), .027	1.73 (0.79–3.81), .157	1.66 (0.76–3.64), .187
T2	78	4.55 (2.59–8.02), <.001	2.97 (1.49–5.91), .004	3.12 (1.51–6.48), .005
T3	147	<.001	<.001	<.001
*P* trend	/	–	–	–

Model 1: Non-adjusted model; Model 2 adjusted for: gender; age; race; Model 3 adjusted for: gender; age; ethnicity; education; BMI; diabetes; physical activity status; alcohol; serum cotinine levels; poverty income ratio; BMI; diabetes; triglycerides; triglyceride; Chromium; Hydrargyrum; Manganese; Plumbum; Selenium.

BLC = blood lead concentration, 95% CI = 95% confidence interval, NHANES = National Health and Nutrition Examination Survey, OR = odds ratio.

### 3.3. Predictive performance of BLC for stroke

**Figure 2.**
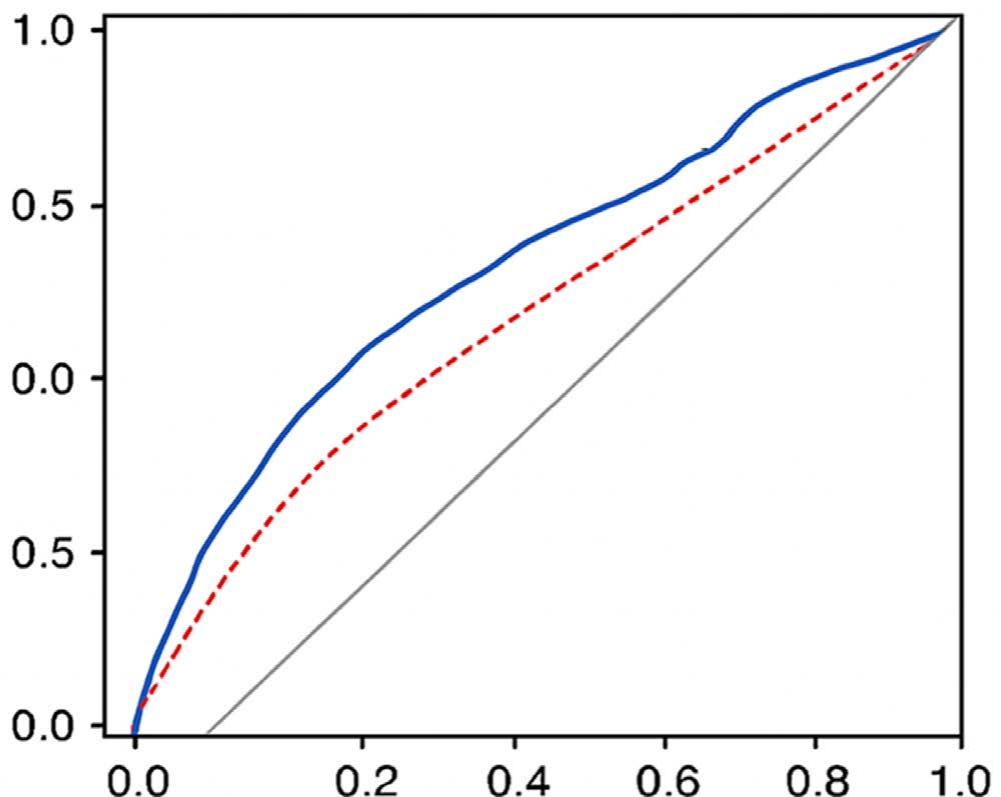
ROC curves comparing the predictive performance of models with and without BLC for stroke. BLC = blood lead concentration, ROC = receiver operating characteristic.

To assess the discriminative ability of BLC for identifying individuals with stroke, we performed a receiver operating characteristic curve analysis (Fig. 2). The area under the receiver operating characteristic curve (area under the curve) for the model including BLC as a continuous variable was 0.68 (95% CI: 0.63–0.73), indicating moderate discriminative ability. When compared to a base model containing only traditional risk factors (age, gender, race/ethnicity, hypertension, diabetes, smoking, and BMI), the addition of BLC significantly improved the area under the curve from 0.65 to 0.68 (*P* = .027), suggesting that BLC provides incremental predictive value beyond conventional risk factors.

As NHANES is a cross-sectional study without follow-up data, survival analyses such as Kaplan–Meier and log-rank tests were not applicable to our study design. However, future longitudinal studies would be valuable to establish the temporal relationship between BLC and stroke incidence.

### 3.4. Stratified and interaction analyses of the association between BLC and stroke among different subgroups of identical covariates

Table 3 showed stratified analyses of the association between BLC and stroke risk. Our findings indicated that the adults with <65 years (OR: 3.12, 95% CI: 1.51–6.46, *P* = .005), female (OR: 2.89, 95% CI: 1.53–5.45, *P* = .003), and non-Hispanic black (OR: 5.39, 95% CI: 1.75–16.57, *P* = .006) had an increased risk of stroke as the concentration of blood lead increasing. Besides, in participants with BMI > 25, BLC was associated with a higher risk of prevalent stroke (BMI: 25–30, OR: 5.55, 95% CI: 1.48–20.74, *P* = .014; BMI > 30, OR: 4.04, 95% CI: 1.47–11.11, *P* = .01). Further analysis revealed a significant interaction of age, ethnicity, serum cotinine, daily alcohol drinking status, and educational level with BLC on stroke risk (*P* for interaction < .05). Among additional covariates in Model 3, there were significant interactions of BLC with plasma triglycerides, history of diabetes, cadmium, and selenium (*P* for interaction < .05) to the risk of stroke. The result suggested that these significant interactions could partly explain why BLC was associated with a reduced risk of stroke in Model 2 and an increased risk of stroke in Model 3.

**Table 3:** Subgroup analysis of associations between BLC and stroke (n = 5013), NHANES 2017–2018.

Quartiles of BLC, µmol/L	Full adjustment model OR (95% CI), *P*		
	Tertile 1	Tertile 2	Tertile 3
	Q1 (<0.03)	Q2 (0.03–0.05)	Q3 (≥0.05)
Stratified by age group (yr)*			
≤65	Reference	2.07 (0.84–5.06), .105	3.12 (1.51–6.46), .005
>65	Reference	0.76 (0.21–2.7), .647	1.75 (0.54–5.65), .323
Stratified by gender†			
Male	Reference	1.26 (0.25–6.45), .769	3.22 (0.69–15.01), .126
Female	Reference	1.87 (1.01–3.46), .048	2.89 (1.53–5.45), .003
Stratified by race/ethnicity‡			
Hispanic	Reference	0.7 (0.22–2.26), .531	1.38 (0.39–4.87), .592
Non-Hispanic White	Reference	1.65 (0.5–5.45), .388	3.13 (0.99–9.89), .052
Non-Hispanic Black	Reference	1.9 (0.58–6.14), .264	5.39 (1.75–16.57), .006
Non-Hispanic Black	Reference	0.42 (0.03–5.86), .492	Empty
Other race§	Reference	59.27 (4.96–708.34), .003	17.82 (0.87–367.18), .061
Stratified by BMI∥			
<20	Reference	0.13 (0–2194.59), .66	71.25 (0–1.13e + 07), .46
20–25	Reference	1.61 (0.43–6.01), .456	0.91 (0.22–3.74), .886
25–30	Reference	1.84 (0.23–14.4), .538	5.55 (1.48–20.74), .014
>30	Reference	2.06 (0.76–5.55), .142	4.04 (1.47–11.11), .01

95% CI = 95% confidence interval, BLC = blood lead concentration, BMI = body mass index, NHANES = National Health and Nutrition Examination Survey, OR = odds ratio.

* After adjusting for gender; ethnicity; education; BMI; diabetes; physical activity status; alcohol; serum cotinine levels; poverty income ratio; BMI; diabetes; triglycerides; triglyceride; Chromium; Hydrargyrum; Manganese; Plumbum; Selenium.

† After adjusting for age; ethnicity; education; BMI; diabetes; physical activity status; alcohol; serum cotinine levels; poverty income ratio; BMI; diabetes; triglycerides; triglyceride; Chromium; Hydrargyrum; Manganese; Plumbum; Selenium.

‡ After adjusting for age, gender; education; BMI; diabetes; physical activity status; alcohol; serum cotinine levels; poverty income ratio; BMI; diabetes; triglycerides; triglyceride; Chromium; Hydrargyrum; Manganese; Plumbum; Selenium.

§ Other races include Asian, American Indian or Alaska Native, Native Hawaiian or other Pacific Islander, and multiracial persons.

∥ After adjusting for gender; ethnicity; education; diabetes; physical activity status; alcohol; serum cotinine levels; poverty income ratio; BMI; diabetes; triglycerides; triglyceride; Chromium; Hydrargyrum; Manganese; Plumbum; Selenium.

## 4. Discussion

Our study makes several important contributions to the literature on environmental exposures and stroke risk. Using nationally representative data, we demonstrate a significant positive association between BLC and stroke prevalence in the U.S. adult population, identify important demographic modifiers of this relationship, and provide evidence that even low-level lead exposure may contribute to cerebrovascular disease risk. These findings highlight the potential public health benefits of further reducing population lead exposure.

This study extends previous work on this topic in several ways. The study conducted by Cao et al indicates a positive correlation between lead exposure and stroke mortality, thereby suggesting that lead may be a risk factor.^[13]^ Given that the vast majority of their study population was male and there was a lack of data regarding factors such as smoking, their results should be interpreted with caution. Our research, after adjusting for multiple covariates related to stroke, discovered that BLC still presented statistically significant associations with stroke occurrence across different demographic groups. Furthermore, while there is sufficient evidence to infer a causal relationship between lead exposure and hypertension, the connection between blood lead levels and stroke incidence lacks sufficient evidence to establish causality.^[14]^ The results of the study conducted by Gump BB et al indicate that low blood lead levels during the prenatal and early childhood periods are associated with alterations in cardiovascular function, particularly with elevated systolic blood pressure and vascular resistance.^[15]^ Even at lead levels below the threshold, lead exposure may still exert adverse effects on cardiovascular health. The study by ***Harari F et al demonstrated that long-term lead exposure increases the risk of atherosclerosis.^[16]^

While reviewing the literature, we came across an intriguing case. A middle-aged male with a history of recurrent abdominal pain and limb weakness due to a retained bullet was treated with an oral penicillin chelating agent.^[17]^ Despite successful reduction in BLC and symptom improvement, the patient later developed subarachnoid hemorrhage. This highlights the importance of monitoring patients with lead exposure history. Additionally, we observed a stronger correlation between BLC and stroke than with other traditional risk factors, emphasizing the necessity for further investigation into this relationship.

Our findings reinforce observations highlighting the potential link between BLC and stroke occurrence. However, several other studies have reported differing results. Kim M et al found no significant correlation between BLC and stroke in their general population sample, though they observed an association among individuals with hypertension in the Korean population.^[14]^ Another prospective cohort study examining the impact of blood lead on stroke mortality in older women found no variation in risk based on BLC. These discrepancies likely reflect heterogeneity in study populations across gender, age, geographical location, race, and ethnicity.

We hypothesize several potential mechanisms underlying the association between elevated BLCs and stroke: Oxidative stress: Lead exposure induces reactive oxygen species generation, damaging endothelial cells and promoting arteriosclerosis^[18]^; Inflammatory response: Lead activates immune responses and increases pro-inflammatory factors, causing vascular dysfunction and promoting small vessel disease^[19,20]^; and Hypertension and haemorheological alterations: Lead exposure affects kidney function, endothelial systems, platelet aggregation, and blood viscosity, all contributing to stroke risk.^[21]^

This study has several limitations. The cross-sectional design precludes establishing temporal relationships between BLC and stroke. The survey data did not distinguish between ischemic and hemorrhagic stroke subtypes. Information regarding participants’ control status of systemic diseases was limited, though these conditions are typically stable long-term. Finally, while self-reported stroke diagnosis in NHANES lacks extensive validation, previous epidemiological studies report high sensitivity and specificity for such self-reporting, supporting the validity of our outcome assessment.

## 5. Conclusion

Our findings indicated that the potentially harmful effects of BLC on stroke in individuals. The associations persisted in the sensitivity analysis. Screening for serum lead concentrations may be regarded as a prophylactic measure for individuals with an elevated risk of stroke, given that lead exposure confers no health benefits. However, large-scale prospective cohort studies are required to identify the causality between BLC and the risk of stroke.

## Acknowledgments

We wish to thank the NHANES program for their effort in data collection and sharing.

## Author contributions

**Conceptualization**: Jian Li.

**Data analysis**: Jian Li.

**Data interpretation**: Jian Li, Qiujing Li, Lichun Zhou.

**Writing – original draft:** Jian Li.

**Writing – review & editing:** Qiujing Li, Lichun Zhou.

**Approval of the final manuscript**: Jian Li, Qiujing Li, Lichun Zhou.
